# Persistent Diarrhea Due to Cyclospora cayetanensis Diagnosed by Multiplex GI PCR in a Patient With HIV: A Case Report

**DOI:** 10.7759/cureus.116251

**Published:** 2026-09-14

**Authors:** Deepak Sukumar, Brian A Moreno, Samiksha Chemukula

**Affiliations:** 1 Emergency Medicine, Broward Health, Fort Lauderdale, USA; 2 Dermatology, Lake Erie College of Osteopathic Medicine, Bradenton, USA; 3 Biology, Nova Southeastern University, Fort Lauderdale, USA

**Keywords:** bactrim ds, cyclospora, cyclospora cayetanensis, gastrointestinal infection, infectious diarrhea

## Abstract

*Cyclospora cayetanensis*is a food- and waterborne coccidian parasite that causes prolonged watery diarrhea. Seasonal outbreaks and the potential for delayed diagnosis highlight the importance of recognizing this pathogen in patients presenting with persistent diarrhea. We present the case of a 49-year-old male with a history of HIV receiving bictegravir/emtricitabine/tenofovir alafenamide, type 2 diabetes mellitus, and hypertension who presented with three weeks of persistent watery diarrhea. He had previously been evaluated in the ED and discharged on ciprofloxacin and dicyclomine after a conventional bacterial stool culture did not identify an enteric pathogen, but his symptoms persisted. On repeat presentation, his vital signs were stable, and physical examination was unremarkable. A multiplex GI PCR panel detected *C. cayetanensis*. The patient received IV hydration and was discharged in stable condition with trimethoprim-sulfamethoxazole (TMP-SMX) 160/800 mg twice daily for 10 days. This case highlights the diagnostic delay that can occur when conventional bacterial stool culture is unrevealing and empiric antibiotic therapy fails to improve persistent diarrhea. Broader testing with multiplex GI PCR identified *C. cayetanensis*, allowing targeted therapy with TMP-SMX. The educational value lies in recognizing when to broaden diagnostic testing for a treatable parasitic infection after an inconclusive initial evaluation.

## Introduction

*Cyclospora cayetanensis *is an obligate intracellular coccidian protozoan and the only species of the genus known to infect humans. Infection occurs through ingestion of sporulated oocysts in contaminated food or water. Following excystation in the GI tract, released sporozoites invade intestinal epithelial cells and may cause prolonged watery diarrhea, anorexia, abdominal cramping, nausea, fatigue, and weight loss. Although infection may be self-limited in immunocompetent individuals, a more prolonged or severe clinical course has been described in patients with impaired cellular immunity, including those with advanced or inadequately controlled HIV infection. Extraintestinal manifestations, including biliary tract disease, have also been reported in patients with advanced HIV/AIDS [1,2].

Diagnosis may be delayed because conventional bacterial stool cultures do not detect *C. cayetanensis*. Identification requires parasite-directed testing, specialized microscopy, or a molecular assay that includes the organism. Multiplex GI PCR panels have expanded the ability to detect bacterial, viral, and parasitic enteric pathogens within a single test and may facilitate earlier diagnosis and targeted antimicrobial therapy [3,4].

We present the case of a patient living with HIV who developed three weeks of persistent watery diarrhea and remained symptomatic after an initial evaluation with an unrevealing conventional bacterial stool culture and empiric ciprofloxacin therapy. A multiplex GI PCR panel subsequently detected *C. cayetanensis*, allowing initiation of targeted treatment with trimethoprim-sulfamethoxazole (TMP-SMX). This case highlights the diagnostic limitations of conventional stool culture and the value of molecular testing in patients with persistent diarrheal illness.

## Case presentation

A 49-year-old male with a medical history significant for HIV, type 2 diabetes mellitus, and hypertension presented to the ED with three weeks of persistent watery diarrhea. He was receiving bictegravir/emtricitabine/tenofovir alafenamide for HIV infection. A detailed epidemiologic history, including recent travel, food or untreated-water exposures, restaurant meals, sick contacts, occupational exposures, and known outbreak exposure, was not available in the source documentation. One week prior, he had presented to the ED with similar symptoms. At that time, a conventional bacterial stool culture did not identify an enteric bacterial pathogen, and he was discharged with ciprofloxacin and dicyclomine. The rationale for empiric ciprofloxacin at the initial visit was not documented in the source information available for this report. Despite completing the prescribed treatment, his symptoms persisted without improvement.

On repeat presentation, he was hemodynamically stable, with a heart rate of 68 beats per minute and a blood pressure of 121/76 mmHg. His respiratory rate was mildly elevated at 22 breaths per minute. Physical examination was otherwise unremarkable.

Given his persistent symptoms and history of HIV infection, a multiplex GI PCR panel was obtained and detected *C. cayetanensis*. The available microbiologic testing is summarized in Table 1. Additional numerical laboratory values, including a contemporaneous CD4 count and HIV viral load, were not available in the clinical information used for this report. No imaging results were available in the source clinical information; therefore, imaging findings are not reported.

**Table 1:** Available microbiologic testing.

Test	Result	Expected/reference result
Conventional bacterial stool culture	No enteric bacterial pathogen identified	No enteric bacterial pathogen identified
Multiplex GI PCR	*Cyclospora cayetanensis *detected	Not detected

The patient received IV fluid resuscitation in the ED and was prescribed TMP-SMX 160/800 mg orally twice daily for 10 days. Following IV hydration and clinical improvement in the ED, he was discharged home with TMP-SMX and instructions for outpatient follow-up. Post-discharge information regarding treatment adherence, symptom resolution, recurrence, and subsequent healthcare utilization was not available.

## Discussion

*C. cayetanensis* is an enteric coccidian parasite transmitted through ingestion of sporulated oocysts in contaminated food or water. Infection commonly presents with watery diarrhea, abdominal cramping, anorexia, nausea, and constitutional symptoms and may follow a prolonged or relapsing course. In the Northern Hemisphere, cyclosporiasis demonstrates seasonal variation, with an increased incidence during the warmer months. Because freshly excreted oocysts require time in the environment to sporulate and become infectious, direct person-to-person transmission is considered uncommon [1].

The relationship between HIV infection and cyclosporiasis has been described extensively, particularly in earlier literature involving patients with more advanced immune dysfunction. A systematic review by Ramezanzadeh et al. demonstrated an association between HIV infection and *C. cayetanensis* infection, supporting consideration of this organism in patients with HIV who develop persistent diarrheal illness [2]. Earlier clinical studies also illustrate the potentially prolonged course of infection in this population. Pape et al. evaluated 43 patients with HIV and Cyclospora-associated diarrhea and reported rapid clinical response to TMP-SMX; however, recurrent symptomatic infection occurred in 12 of 28 patients (43%) who were followed for at least one month [5]. Sifuentes-Osornio et al. described 12 patients with cyclosporiasis, seven of whom had AIDS. The mean duration of illness in that series was 94 days; patients with AIDS experienced greater weight loss and tended to have a more prolonged illness, and two patients with AIDS developed biliary disease [6].

The present case differs from these earlier reports in several respects. The patient returned for evaluation after three weeks of persistent diarrhea and remained hemodynamically stable at the time of his repeat presentation. However, a contemporaneous CD4 count and HIV viral load were unavailable, preventing assessment of the degree of HIV-related immune dysfunction. Follow-up after treatment was also unavailable, so comparison with the recurrence rates described in earlier HIV-associated cohorts is not possible. These limitations are important because HIV infection alone does not establish the degree of immunosuppression, particularly in a patient receiving antiretroviral therapy.

This case also illustrates an important diagnostic limitation of conventional bacterial stool culture. Routine bacterial culture does not detect *C. cayetanensis*, which requires parasite-directed testing, appropriate microscopy, or a molecular assay that includes the organism [1,3]. In this patient, the initial bacterial stool culture did not identify an enteric pathogen, and symptoms continued despite empiric treatment. Multiplex GI PCR subsequently identified *C. cayetanensis *and provided a specific microbiologic diagnosis. Multiplex GI panels can improve detection of enteric pathogens that might otherwise require separate diagnostic approaches and may shorten the time to targeted treatment [3,4].

The patient’s initial exposure to ciprofloxacin is also notable in the context of previously published treatment data. Verdier et al. conducted a randomized controlled trial involving 42 HIV-infected patients with chronic diarrhea caused by either *C. cayetanensis *or *Isospora belli*. Patients received either TMP-SMX or ciprofloxacin for seven days. Diarrhea resolved in all 19 patients treated with TMP-SMX compared with 20 of 23 patients treated with ciprofloxacin, while negative stool examinations at day seven occurred in 18 of 19 and 16 of 23 patients, respectively. Diarrhea resolved more rapidly with TMP-SMX, leading the investigators to conclude that TMP-SMX was more effective, although ciprofloxacin remained an alternative for patients unable to tolerate TMP-SMX [7]. Because outcomes in that study included both cyclosporiasis and isosporiasis, the findings should not be interpreted as a Cyclospora-only comparison. Nevertheless, they are clinically relevant to the present case, in which symptoms persisted following empiric ciprofloxacin and pathogen-directed therapy was subsequently changed to TMP-SMX after PCR established the diagnosis.

TMP-SMX remains the treatment of choice for cyclosporiasis. A commonly recommended adult regimen is trimethoprim 160 mg with sulfamethoxazole 800 mg orally twice daily for seven to 10 days, although longer treatment may be necessary in some immunocompromised patients [1,3]. The 10-day course prescribed in this case was therefore consistent with established treatment approaches. The absence of post-discharge follow-up prevents determination of the patient’s final clinical response or whether recurrence occurred.

Several limitations of this case should be acknowledged. Numerical laboratory data beyond the available microbiologic testing were not available for inclusion. In particular, the absence of a contemporaneous CD4 count and HIV viral load limits characterization of the patient’s immune status. Additional details regarding stool frequency, associated symptoms, oral intake, and weight change were not available. Imaging results were also unavailable, and post-treatment follow-up was not available to establish symptom resolution, adherence, microbiologic clearance, or recurrence. Despite these limitations, the case demonstrates a clinically relevant diagnostic sequence in which persistent diarrhea after an unrevealing conventional bacterial stool culture prompted molecular testing that identified a treatable parasitic infection.

## Conclusions

*C. cayetanensis* should be considered in patients with persistent watery diarrhea when the initial evaluation is unrevealing, particularly when clinical or epidemiologic factors raise concern for prolonged infection. Conventional bacterial stool culture does not detect Cyclospora, and parasite-specific testing or a multiplex GI PCR panel that includes the organism may be necessary to establish the diagnosis. In this patient with HIV receiving antiretroviral therapy, multiplex PCR identified *C. cayetanensis* after an unrevealing bacterial stool culture and allowed initiation of targeted treatment with TMP-SMX. Earlier studies in patients with HIV demonstrate that cyclosporiasis may be prolonged or recurrent and support TMP-SMX as preferred therapy. Because contemporaneous measures of HIV control and post-treatment follow-up were unavailable in this case, the contribution of immune status and the ultimate clinical outcome could not be determined.
